# Verticalization Therapy for Acute Respiratory Distress Syndrome Patients Receiving Veno-Venous Extracorporeal Membrane Oxygenation

**DOI:** 10.7759/cureus.40094

**Published:** 2023-06-07

**Authors:** Shahriar Shayan, Alexander M DeLeon, Randy McGregor, Thomas Mader, Mia Garino, Christopher Mehta

**Affiliations:** 1 Anesthesiology, Northwestern Memorial Hospital, Chicago, USA; 2 Cardiac Surgery, Northwestern Memorial Hospital, Chicago, USA

**Keywords:** prone, ecmo, hypoxemia, verticalization therapy, ards

## Abstract

Persistent hypoxemia during veno-venous extracorporeal membrane oxygenation (VV-ECMO) for supporting acute respiratory distress syndrome (ARDS) patients is a clinical challenge for intensive care medical providers. Prone positioning is an effective strategy to treat persistent hypoxemia; however, placing a patient in a prone position is resource intensive with significant risks to the patient. We present a patient with severe ARDS receiving VV-ECMO who underwent verticalization therapy and subsequently recovered pulmonary function.

## Introduction

Veno-venous extracorporeal membrane oxygenation therapy (VV-ECMO) is a modality to support patients with acute respiratory distress syndrome (ARDS) when traditional positive pressure ventilation has failed. The institution of VV-ECMO allows the patient to maintain end-organ oxygenation while clinicians prescribe an appropriate lung protective strategy. In patients with inadequate oxygenation despite optimizing the extracorporeal membrane oxygenation (ECMO) settings, prone positioning (PP) has been proposed to improve oxygenation through improved lung mechanics and gas exchange [1,2].

PP is logistically difficult and associated with pressure sore development with potential for severe complications such as inadvertent decannulation or extubation [3]. Verticalization therapy (VT) is the placement of the patient with their head up 45 to 90 degrees without flexion at the hip [4]. Two reports demonstrated improvements in the oxygenation of patients with ARDS, increased lung recruitment, and improved static lung compliance [4,5]. VT has not been reported in conjunction with the use of VV-ECMO. A safe and effective alternative to PP for critically ill VV-ECMO patients with insufficient arterial oxygenation would be a useful therapeutic option for intensivists treating ARDS.

We present the first reported case of VT to improve oxygenation in a VV-ECMO patient with severe ARDS resulting in a successful outcome.

## Case presentation

A 31-year-old 160-pound 5'7" (BMI 25.21 kg/m^2^) woman with no significant past medical history other than attention deficit disorder and anxiety presented to an outside hospital (OSH) with worsening respiratory status. She had been initially treated for influenza as an outpatient, yet her respiratory status declined over seven days at the OSH, requiring mechanical ventilation. She was transferred from the OSH via ambulance for further management and to evaluate the need for ECMO.

**Figure 1:**
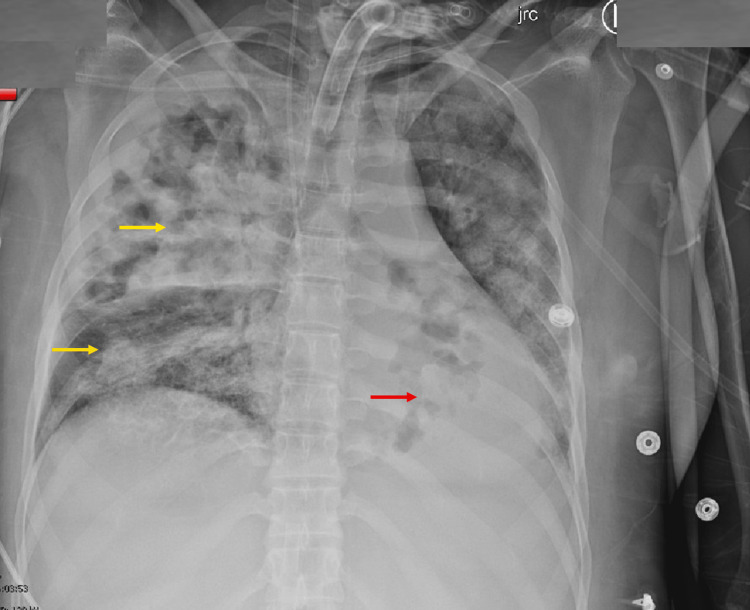
Anterior-Posterior Chest Radiograph on Presentation The chest radiograph demonstrates a dense cavitary right upper lobe pneumonia (yellow arrow), increasing opacities in the right lower lobe (orange arrow), and opacities and dense left lower lobe consolidation (red arrow).

Upon arrival, she was found to have severe ARDS with streptococcal bacterial infection (see Figure 1). Laboratory findings included a procalcitonin level of 32 ng/mL, a white blood cell count of 13 K/UL, a lactate level of 4 mMol/L, a blood culture with gram-positive cocci, and a respiratory culture positive for Streptococcus pneumoniae.

The patient was cannulated for VV-ECMO utilizing a right femoral to right internal jugular vein strategy. In addition to VV-ECMO, she was managed for multifocal Streptococcus pneumonia with multiple antibiotics, including vancomycin, piperacillin-tazobactam, and clindamycin. Once blood cultures confirmed sensitivity to piperacillin-tazobactam, her antibiotic regimen was adjusted. A transthoracic echocardiogram demonstrated a left ventricular ejection fraction of 45 to 50 percent with moderate right ventricular dysfunction and no evidence of endocarditis. The patient was listed as a lung transplant candidate after two weeks in the intensive care unit.

**Figure 2:**
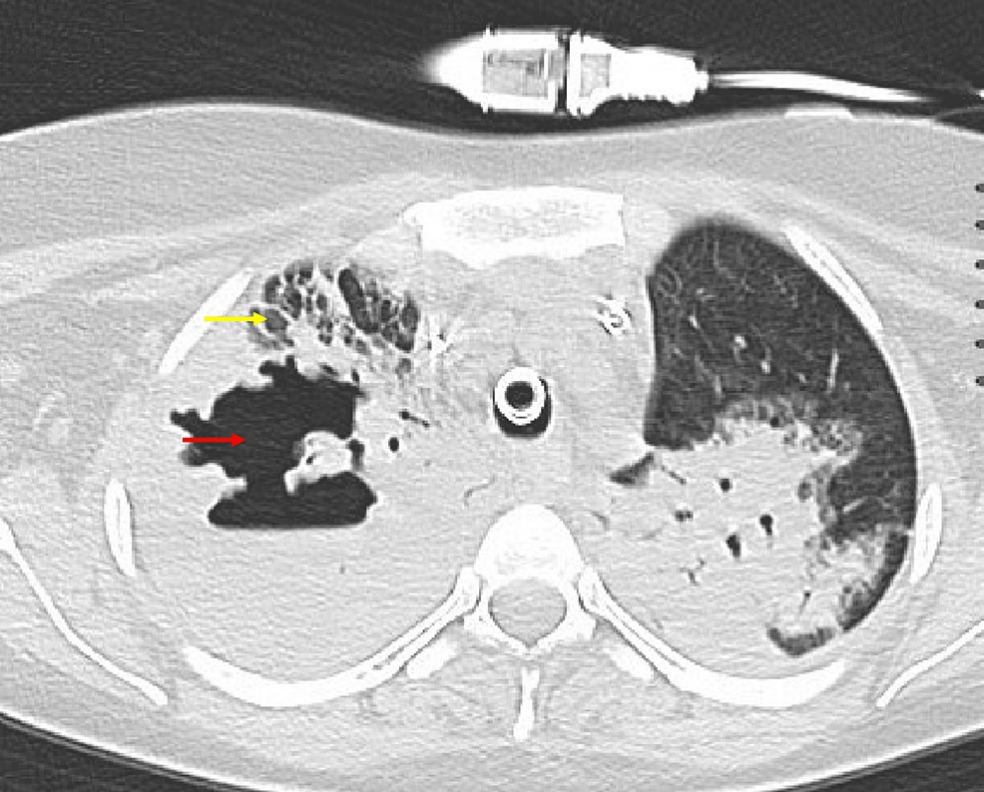
Non-contrast Computed Tomography of the Chest The scan demonstrates complete right upper lobe consolidation (yellow arrow) and a necrotizing cavitary lesion (red arrow).

Computed tomography (CT) of the chest showed severe multifocal consolidation with right upper lung necrotizing lesions (see Figure 2). The patient exhibited continued hypoxemia despite increasing the FDO2 to 100 percent, empiric titration of high positive end-expiratory pressure, pharmacologic muscle relaxation, and lung protective strategies consistent with the principles of the ARDSnet study [6].

Despite the institution of VV-ECMO, the patient experienced a suboptimal improvement in oxygen saturation, irrespective of achieving 5 to 5.5 liters per minute flows. Pre- and post-oxygenator arterial blood gases remained appropriate, demonstrating proper ECMO function. Nitric oxide was initiated on day four post-cannulation, and high-dose methylprednisolone therapy was administered.

**Figure 3:**
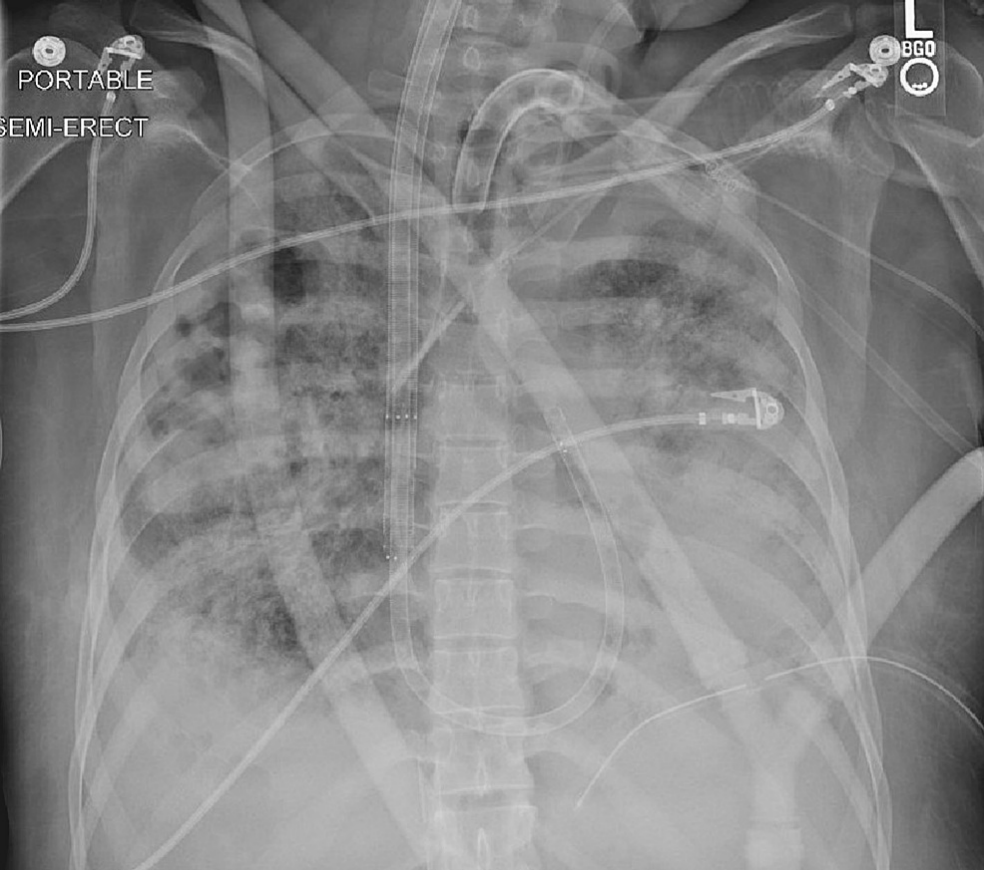
Interval Radiograph One-Month Post-admission Significant diffuse airspace disease is still apparent.

The patient's oxygenation status did not improve; the highest achieved saturation was 88 percent. The chest radiograph showed worsening consolidation (see Figure 3). On post-cannulation day eight, the cannulas were changed to a PROTEK DuoTM (CardiacAssist, Inc., Pittsburgh, PA) cannula system to improve oxygenation by reducing mixing. 

The change in cannula strategy did not significantly improve the patient's oxygenation status; thus, VT was judged as the next appropriate therapeutic option. Therefore, the patient was placed on a CATALYST bed (Kreg Therapeutics, Melrose Park, IL) for 45 to 60 degree-VT. The patient underwent VT twice daily for thirty minutes per session. The hemodynamic profile during VT was stable, with blood pressures of 140/80 mmHg and heart rates of 100 beats per minute on average during VT sessions.

The decisions regarding the duration and degree of VT were based on patient tolerance. Due to deconditioning, achieving more than 60 degrees of VT was limited by her lower extremity weakness. Similarly, sessions longer than 30 minutes were not tolerated due to leg muscle fatigue. During VT sessions, the degree of sedation utilizing infusions of propofol and fentanyl was targeted to a Richmond Agitation-Sedation Scale (RASS) score of zero to negative one, indicating that the patient was alert and calm [7].

**Figure 4:**
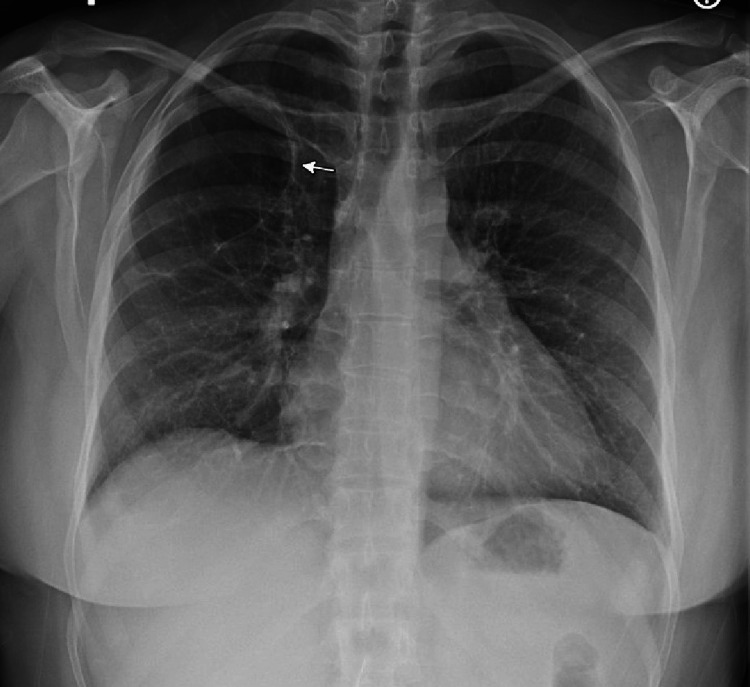
Follow-up Chest Radiograph Two-Weeks Post-ICU Discharge The white arrow indicates a stable large right pneumatocele.

The patient sustained an oxygen saturation above 90 percent for approximately three hours after each VT without changes to the ventilator or ECMO settings. VT sessions continued two to three times daily for one month. As the patient's pulmonary status improved, she was successfully weaned from ECMO and decannulated approximately seven weeks post-cannulation. The patient was discharged from the intensive care unit two weeks later to a long-term acute care hospital. Her chest radiograph two months post-discharge demonstrated a resolution of pneumonia (see Figure 4). On follow-up at two years, the patient reported that her breathing feels "normal" with only mild shortness of breath with strenuous exercise.

## Discussion

Refractory hypoxemia is a treatment problem in patients with severe ARDS who require VV-ECMO. PP has been advocated successfully in VV-ECMO patients to improve oxygenation [1,2]. Giani et al. reported a multicenter cohort study evaluating the efficacy of PP for patients undergoing VV-ECMO for ARDS [1]. Their group reported improved oxygenation, reduced intrapulmonary shunt, and improved hospital mortality.

Given the potential risk for position-related pressure sores or more severe complications such as inadvertent decannulation or extubation, alternatives to PP could be a useful therapeutic option for intensivists caring for ARDS patients [3]. Verticalization for other disease processes, such as refractory intracranial hypertension, has been reported [8]. VT as a ventilation strategy for ARDS patients not receiving ECMO therapy has also been studied. Oxygenation, static compliance, and lung recruitment improvements have been reported with 45 degrees of VT [4,5].

The mechanism for improving oxygenation in VT likely involves improved pulmonary mechanics and improvements in matching ventilation and perfusion. The major risk of VT would be a negative impact on hemodynamics due to worsening preload. Our patient demonstrated a slight reduction in blood pressure and an increased heart rate during VT sessions, yet hemodynamics were stable, and no interventions were necessary for blood pressure management.

VT has not been previously reported for refractory hypoxemia in the setting of ARDS requiring VV-ECMO. We report the first case in the literature of VT to achieve improvement in arterial oxygenation in this setting.

## Conclusions

Managing refractory tissue hypoxemia during VV-ECMO therapy for ARDS patients is a clinical challenge for intensive care physicians. Previously reported proposed changes in patient positioning, such as PP, are effective yet logistically challenging and associated with risks and complications. VT is potentially an effective yet technically less demanding alternative to PP in the setting of VV-ECMO for ARDS patients.
